# Apoptosis and *p53* status predict the efficacy of postoperative administration of UFT in non-small cell lung cancer

**DOI:** 10.1054/bjoc.2000.1579

**Published:** 2001-01

**Authors:** F Tanaka, Y Otake, K Yanagihara, T Yamada, R Miyahara, Y Kawano, M Li, K Inui, H Wada

**Affiliations:** Department of Thoracic Surgery, Faculty of Medicine, Kyoto University, Shogoin-kawahara-cho 54, Sakyo-ku, Kyoto, 606-8397, Japan

**Keywords:** *p53*, *bcl-2*, apoptosis, adjuvant therapy, 5-fluorouracil, UFT

## Abstract

To examine whether efficacy of postoperative oral administration of UFT, a 5-fluorouracil derivative chemotherapeutic agent, may be influenced by incidence of apoptosis (apoptosis index) or apoptosis-related gene status (p53 and bcl-2) of the tumour, a total of 162 patients with pathologic stage I non-small cell lung cancer were retrospectively reviewed. UFT was administrated postoperatively to 44 patients (UFT group), and not to the other 118 patients (Control group). For all patients, 5-year survival rate of the UFT group (79.9%) seemed higher than that of the Control group (69.8%), although without significant difference (P = 0.054). For patients with higher apoptotic index, 5-year survival rate of the UFT group (83.3%) was significantly higher than that of the Control group (67.6%, P = 0.039); for patients with lower apoptotic index, however, there was no difference in the prognosis between these two groups. Similarly, UFT was effective for patients without p53 aberrant expression (5-year survival rates: 95.2% for the UFT group and 74.3% for the Control group, P = 0.022), whereas not effective for patients with p53 aberrant expression. Bcl-2 status did not influence the efficacy of UFT. In conclusion, apoptotic index and p53 status are useful factors to predict the efficacy of postoperative adjuvant therapy using UFT. © 2001 Cancer Research Campaign http://www.bjcancer.com


*British Journal of Cancer* (2001) **84**(2), 263–269
© 2001 Cancer Research Campaign
doi: 10.1054/ bjoc.2000.1579, available online at http://www.idealibrary.com on IDEAL®

http://www.bjcancer.com

### Apoptosis and *p53* status predict the efficacy of postoperative administration of UFT in non-small cell lung cancer

**F Tanaka, Y Otake, K Yanagihara, T Yamada, R Miyahara, Y Kawano, M Li, K Inui and H Wada**

Department of Thoracic Surgery, Faculty of Medicine, Kyoto University; Shogoin-kawahara-cho 54, Sakyo-ku, Kyoto, 606-8397, Japan

**Summary** To examine whether efficacy of postoperative oral administration of UFT, a 5-fluorouracil derivative chemotherapeutic agent, may be influenced by incidence of apoptosis (apoptosis index) or apoptosis-related gene status (*p53* and *bcl-2*) of the tumour, a total of 162 patients with pathologic stage I non-small cell lung cancer were retrospectively reviewed. UFT was administrated postoperatively to 44 patients (UFT group), and not to the other 118 patients (Control group). For all patients, 5-year survival rate of the UFT group (79.9%) seemed higher than that of the Control group (69.8%), although without significant difference ($P$ = 0.054). For patients with higher apoptotic index, 5-year survival rate of the UFT group (83.3%) was significantly higher than that of the Control group (67.6%, $P$ = 0.039); for patients with lower apoptotic index, however, there was no difference in the prognosis between these two groups. Similarly, UFT was effective for patients without *p53* aberrant expression (5-year survival rates: 95.2% for the UFT group and 74.3% for the Control group, $P$ = 0.022), whereas not effective for patients with *p53* aberrant expression. *Bcl-2* status did not influence the efficacy of UFT. In conclusion, apoptotic index and *p53* status are useful factors to predict the efficacy of postoperative adjuvant therapy using UFT. © 2001 Cancer Research Campaign  http://www.bjcancer.com

**Keywords:** *p53*; *bcl-2*; apoptosis; adjuvant therapy; 5-fluorouracil; UFT

Non-small cell lung cancer (NSCLC) is a malignant tumour with poor prognosis, and the postoperative survival is not satisfactory (Mountain, 1997). Although adjuvant therapy was introduced to improve the postoperative prognosis, it has been concluded that radiotherapy can not improve the survival although it can reduce the local recurrence rate. Therefore, systemic chemotherapy has been expected to improve the postoperative survival by suppressing occurrence of distant metastasis, and a variety of chemotherapeutic regimens have been attempted. However, the efficacy of postoperative adjuvant chemotherapy has not been established yet (Ihde et al, 1994).

However, a recent retrospective study conducted by Kyoto University suggested that UFT administration was effective as a postoperative adjuvant therapy for NSCLC (Tanaka et al, 1998a). UFT is an oral 5-fluorouracil (5-FU) derivative drug composed of tegafur (1-[2-tetrahydrofuryl]-5-fluoroiracil, FT) and uraci (U); tegafur is a pro-drug that persistently releases 5-FU, and uracil is added to inhibit degradation of the released 5-FU (Fujii et al, 1978; Ikenaka et al, 1979). As a result, when UFT is administrated, certain 5-FU concentration in blood and tumour tissues can be maintained for a long time. The efficacy of postoperative oral administration of UFT was confirmed by a prospective randomized study conducted by 'The West Japan Study Group for lung cancer surgery'; 5-year survival rates of pathologic (p-) stage I–III, NSCLC were 64.1%, 60.6% and 49.0% in patients who received postoperative UFT administration (400 mg body$^{-1}$ day$^{-1}$ for one year after surgery, UFT group), in those who received the same UFT administration following intravenous infusion of CDDP (50 mg/m$^2$ body surface for one course) + VDS (2–3 mg kg body weight$^{-1}$ for 3 courses) (CVUft group), and in those who received no adjuvant therapy (Control group), respectively ($P$ = 0.044 among 3 groups, and $P$ = 0.019 between the UFT group and the Control group) (Wada et al, 1996). The efficacy of postoperative UFT administration in NSCLC was demonstrated in other prospective randomized studies (The Study Group of Adjuvant Chemotherapy for Lung Cancer (Chubu, Japan), 1995; Wada et al, 1999). Although these results suggest the efficacy of postoperative UFT administration, postoperative recurrence may appear in some cases even if UFT administration is performed. Thus, if whether UFT administration is effective or not can be predicted in an individual patient before the administration, postoperative prognosis can be improved when efficacy of UFT administration is expected, and medical and financial disadvantage of UFT administration can be relieved when the efficacy is not expected.

The *p53* tumour suppressor gene protects the genome against a variety of damages, and suppresses occurrence of malignant tumour (Levine et al, 1991). The *p53* gene induces apoptosis as well as regulates the cell cycle (Clarke et al, 1993; Lowe et al, 1993). Apoptosis is a kind of cell death distinct from necrosis, showing morphological features such as cell shrinkage, loss of cell–cell contact, chromatin condensation and intranucleosomal degradation of DNA (Kerr et al, 1972). Apoptosis is an essential phenomenon for normal development and maintenance of homeostasis, and also plays an important role in suppressing proliferation of malignant tumour cells (Symonds et al, 1994; Holmgren et al, 1995). It is known that apoptosis is induced by various

*Received 24 August 2000*
*Revised 9 October 2000*
*Accepted 18 October 2000*

*Correspondence to:* H Wada

Parts of this article was presented at the 34th annual meeting of ASCO (Los Angeles, CA, abstract No. 2110) and the 35th annual meeting of ASCO (Atlanta, GA, abstract No. 2393).

anticancer agents and radiotherapy. Therefore, it has been reported that sensitivity to anticancer agents and radiotherapy can be influenced by the status of some genes associated with apoptosis such as the *p53* gene (Clarke et al, 1993; Lowe et al, 1993).

In a preliminary study, we have already reported that the efficacy of oral administration of FT and UFT after surgery for NSCLC may be influenced by *p53* status (Tanaka et al, 1999a). Moreover, we have already reported that balance between incidence apoptotic cell death and tumour-cell proliferation is an important factor to predict postoperative survival in resected NSCLC (Tanaka et al, 1999b). In the present study, whether the efficacy of postoperative UFT administration was influenced by incidence of apoptosis (apoptotic index: AI) and by status of *p53* and *bcl-2* regulating apoptosis in more homogeneous patients (p-stage I patients) was examined.

### PATIENTS AND METHODS

A total of 163 consecutive patients with p-stage I, NSCLC who underwent complete tumour resection and mediastinal lymph node dissection without any preoperative therapy at the Department of Thoracic Surgery, Kyoto University between January 1, 1987 and December 31, 1992, were reviewed. P-stage and histological typing were re-evaluated and determined according to the current TNM classification (Mountain, 1997) and the current classification by World Health Organization (Travis et al, 1999), respectively. One patient was excluded from the study due to operation-related death, and thus a final total of 162 patients (122 males and 40 females) were evaluated. For all these patients, the inpatient medical records, chest X-ray films, whole-body CT films, bone and gallium scanning data and records of surgery were reviewed without knowledge of the results of immunohistochemical staining. Follow-up of the postoperative clinical course was conducted by outpatient medical records and by inquiries by telephone or letter. The day of thoracotomy was considered the starting day for counting postoperative survival days.

### Clinical characteristics of patients

UFT was administrated to all patients in whom an informed consent had been taken; UFT was not administrated if not. As a result, among all 162 patients, UFT (Taiho Pharmaceutical Co, Tokyo, Japan) was administered to 44 patients (UFT group), and not administered to the other 118 patients (control group). Administration dose of UFT was 300 mg day$^{-1}$ body$^{-1}$ (body weight < 50 kg) or 400 mg day$^{-1}$ body$^{-1}$ (body weight ≥50 kg). Oral administration of UFT was initiated within one month after surgery. UFT was administered for at least one year if the patients were alive; for deceased patients, UFT was administered until oral administration became impossible. There were no significant differences in clinical characteristics of patients between the UFT group ant the control group (Table 1). No other preoperative, intraoperative, or postoperative therapy was performed in any case.

### Tissue preparation

Detection of apoptotic cells was performed with the terminal deoxynucleotidyl transferase-mediated dUTP-biotin nick end-labelling (TUNEL) method. Expression of PCNA (proliferating cell nuclear antigen), which was expressed in nucleus at the late G1 phase and S phase of the cell cycle, was examined immunohistochemically as an index of cell proliferation. Aberrant expression of *p53* and expression of *bcl-2* were also examined immunohistochemically. Serial 4-μm sections were prepared from each formalin-fixed and paraffin-embedded tumour specimen, and served for routine haematoxylin and eosin (H&E) staining, the TUNEL staining and immunohistochemical staining (IHS). Dewaxed sections were digested with 20 μg ml$^{-1}$ proteinase K (Boehringer Manheim, Manheim, Germany) for 20 minutes at 25˚C for the TUNEL staining, and were heated in a microwave oven for 5 minutes three times for IHS. Endogenous peroxidase was inactivated by incubating the sections with 0.03% $H_2O_2$ in methanol for 30 minutes at 25˚C. To reduce unspecific labelling, the sections were incubated with normal calf serum (DAKO Japan, Kyoto, Japan).

### Detection of apoptosis

The TUNEL staining was performed using In Situ Death Detection Kit, POD (Boehringer Manheim, Manheim, Germany) following the manufactured protocol as described previously (Tanaka et al, 1999b). The specificity of the TUNEL staining of apoptotic cells was confirmed by making the negative and the positive control slides at every staining. As negative control slides, sections incubated with the TUNEL reaction mixture without TdT were used. As positive control slides, sections treated with DNase 1 (0.7 mg ml$^{-1}$, Stratgene, La Jolla, CA) for 10 minutes at 25˚C before the TUNEL reaction were used. Apoptotic cells were determined with careful observation of TUNEL-staining sections and serial H&E-staining sections. TUNEL-positive staining cells that represent histological features of necrosis in H&E-staining sections were not considered to be apoptotic cells. In each case, a total of 10 000 tumour cells, 1000 tumour cells each in 10 different fields, were evaluated at high magnification (×400) by two authors independently (F.T. and Y.O) without knowledge of clinical data. When a different evaluation of apoptotic cells was made, the field was re-evaluated until the evaluation coincided. The apoptotic index (AI) was defined as the number of apoptotic cells per 1000 tumour cells.

### IHS

Procedure of IHS using streptoavidin-biotinylated horseradish-peroxidase complex method (LSAB kit; DAKO JAPAN, Kyoto, Japan) was described previously (Tanaka et al, 1998b, 1999a, 1999b). Mouse anti-human PCNA, monoclonal antibody (MoAb) PC-10 (mouse IgG2a, kappa 400 μg ml$^{-1}$ DAKO Japan) diluted at 1:50, anti-human *p53* MoAb, clone DO-7 (mouse IgG2b, kappa, 250 μg ml$^{-1}$, DAKO Japan) diluted at 1:50, and anti-human *bc1-2* MoAb, clone 124 (mouse IgG, kappa, 200 μg ml$^{-1}$, DAKO Japan) diluted at 1:50 and mouse were used as the primary antibody. Stained tissue slides were evaluated by two of the authors (F.T. and Y.O.) independently without knowledge of clinical data. A total of 1000 tumour cells were counted, and the percentages of positive cells were determined. When the percentage of positive-staining cells exceeded 5%, the slide was judged to exhibit aberrant expression of *p53* or positive expression of *bcl-2*. The fraction of proliferative cells was represented by the percentage of PCNA-positive tumour cells (proliferative index: PI).

### Statistical methods

Counts were compared by the chi-square test, and trends in counts were analysed by the chi-square test for trends. Continuous data

© 2001 Cancer Research Campaign were compared using Student's *t*-test if the distribution of samples was normal, or using Mann-Whitney U-test if the sample distribution was asymmetrical. Postoperative survival rate was analysed by the Kaplan-Meier method, and the difference was assessed by the log-rank test. Multivariate analysis of prognostic factors was performed using Cox's regression model. Differences were considered significant when *P*- value was less than 0.05. All statistical manipulations were performed using the SPSS for Windows software system (SPSS Inc, Chicago, IL, USA, 1993).

### RESULTS

### Incidence of apoptosis (AI), aberrant expression of *p53* and expression of *bcl-2*

The mean AI for all patients was 19.2 ± 2.0 (mean ± standard error: SE), and the median AI was 10.9. There was no difference in the mean AI between the UFT group and the Control group (Table 1). Even if the patients were divided to patients with higher AI (AI ≥ 10.9) and those with lower AI (AI < 10.9), there was no difference in ratios of higher and lower AI patients between the UFT group and the Control group. In 67 of 162 (41.4%) patients, *p53* aberrant expression was demonstrated. In only 33 (20.4%) patients, *bcl-2* was expressed. There was no significant difference in ratios of patients with *p53* aberrant expression or in ratios of *bcl-2*-positive patients between the UFT group and the Control group (Table 1).

### Correlation of AI with cell proliferation, aberrant expression of *p53* and expression of *bcl-2*

Correlation between AI and fraction of proliferative cancer cells (PI) was examined (Table 2). The mean PI in higher-AI patients was 53.5%, which was significantly higher than that in lower-AI patients (31.6%). Moreover, the number of patients showing higher PI (PI ≥ 44%) was larger in higher-AI patients than in lower-AI patients. These results demonstrated that more cancer cells showing active proliferation have more chances of undergoing apoptosis. On the other hand, no significant correlation of AI with *p53* aberrant expression or *bcl-2* expression was observed.

**Table 2** Correlation between apoptotic index (AI) and apoptosis-related gene (*p53*, *bcl-2*) status

| | Lower-AI[b] | Higher-AI[b] | *P* value[a] |
|---|---|---|---|
| Proliferative index (PI, mean) | 31.6% | 53.5% | <0.001 |
| Lower-PI[c] | 58 (71.6%) | 24 (29.6%) | <0.001 |
| Higher-PI[c] | 23 (28.4%) | 57 (70.4%) | |
| *p-53* aberrant expression | | | |
| Negative | 50 (61.7%) | 45 (55.6%) | 0.425 |
| Positive | 31 (38.3%) | 36 (44.4%) | |
| *bcl-2* expression | | | |
| Negative | 62 (76.5%) | 67 (82.7%) | 0.329 |
| Positive | 19 (23.5%) | 14 (17.3%) | |

[a]*P* value: Comparison of patients' characteristics between patients with Lower-AI and those with Higher-AI. [b]Lower-AI: AI < 10.9 (median AI). Higher-AI: AI ≥ 10.9 (median AI). [c]Lower-PI: PI < 44% (median PI). Higher-PI: PI ≥ 44% (median PI).

### AI, aberrant expression of *p53* expression of *bcl-2* and postoperative prognosis

According to univariate analysis of prognostic factors, 5-year survival rates of patients with and those without *p53* aberrant expression were 60.4% and 80.1%, suggesting *p53* aberrant expression was a significant factor to predict poor prognosis (*P* = 0.011). In contrast, no significant difference was observed in the prognosis between patients with and those without *bcl-2* expression, or between patients with higher AI and those with lower AI (Table 3). Multivariate analysis also confirmed that *p53* aberrant

**Table 1** Characteristics of patients, apoptotic index (AI) and apoptosis-related gene (p53, bcl-2) status in patients with and without postoperative oral administration of UFT

| | All patients | Patients with UFT administration | Patients without UFT administration | *P* value[a] |
|---|---|---|---|---|
| Gender Male | 122 (75.3%) | 31 (70.5%) | 91 (77.1%) | 0.382 |
| Female | 40 (24.7%) | 13 (29.5%) | 27 (22.9%) | |
| Age (mean, years) | 62.7 | 61.8 | 63.0 | 0.460 |
| Performance status 0 | 147 (90.7%) | 40 (90.9%) | 107 (90.7%) | 0.755 |
| 1 | 15 (9.3%) | 4 (9.1%) | 11 (9.3%) | |
| Pathologic T1 (stage IA) | 84 (51.9%) | 23 (52.3%) | 61 (51.7%) | 0.948 |
| T2 (stage IB) | 78 (48.1%) | 21 (47.7%) | 57 (48.3%) | |
| Histology Squamous cell | 55 (34.0%) | 14 (31.8%) | 41 (34.7%) | |
| Adenocarcinoma | 93 (57.4%) | 27 (61.4%) | 66 (55.9%) | 0.713 |
| Large cell | 11 (6.8%) | 2 (4.5%) | 9 (7.6%) | |
| Others | 3 | 1 | 2 | |
| Apoptotic index (AI, mean) | 19.2/1000 cancer cells | 20.8 | 18.6 | 0.637 |
| Lower-AI (<10.9) | 81 (50.0%) | 23 (52.3%) | 58 (49.2%) | 0.724 |
| Higher-AI (≥10.9) | 81 (50.0%) | 21 (47.7%) | 60 (50.8%) | |
| *p53* aberrant expression | | | | |
| Negative | 95 (58.6%) | 25 (56.8%) | 70 (59.3%) | 0.773 |
| Positive | 67 (41.4%) | 19 (43.2%) | 48 (40.7%) | |
| *bcl-2* expression | | | | |
| Negative | 129 (79.6%) | 32 (72.7%) | 97 (82.2%) | 0.183 |
| Positive | 33 (20.4%) | 12 (27.3%) | 21 (17.8%) | |

[a]*P* value: Comparison of patients' characteristics between patients with and without postoperative administration of UFT.

© 2001 Cancer Research Campaign

**Table 3** Unvariate analysis of prognostic factors

| Prognostic factors | 5-year survival rate | *P* value |
|---|---|---|
| Gender | | |
| Male/Female | 66.9%/82.7% | 0.467 |
| Age[a] | | |
| Lower age / Higher age | 76.3%/64.7% | 0.086 |
| Performance Status | | |
| 0 / 1 | 72.0%/46.2% | 0.124 |
| Pathologic-T factor | | |
| T1 (IA) / T2 (IB) | 71.9%/69.4% | 0.758 |
| Histology | | |
| Squamous cell / Adenocarcinoma | 67.9%/73.1% | 0.909 |
| *p53* aberrant expression | | |
| Negative / Positive | 80.1%/60.4% | 0.011 |
| *bcl-2* expression | | |
| Negative / Positive | 71.2%/75.9% | 0.349 |
| Apoptotic index (AI)[b] | | |
| Lower / Higher | 72.1%/71.5% | 0.684 |
| Proliferative index (PI)[c] | | |
| Lower/Higher | 74.0% / 75.6% | 0.261 |
| Postoperative UFT administration | | |
| No / Yes | 79.9% / 69.8% | 0.054 |

[a]Lower age: Age < 64.0 years (median age). Higher age: Age ≥ 64.0 years (median age). [b]Lower-AI: AI < 10.9 (median AI). Higher-AI: AI ≥ 10.9 (median AI). [c]Lower-PI: PI < 44% (median PI). Higher-PI: PI ≥ 44% (median PI).

expression was a significant factor to predict poor postoperative, and that *bcl-2* expression or AI could not be a significant prognostic factor (Table 4).

### Efficacy of postoperative UFT administration and AI, aberrant expression of *p53* and expression of *bcl-2*

5-year survival rates of the UFT group and the Control group were 79.9% and 69.8%, respectively. Although the UFT group seemed to show favourable prognosis as compared with the Control group, the difference proved not be significant ($P = 0.054$, Figure 1).

The efficacy of the UFT administration was compared between patients without and those with *p53* aberrant expression. In patients without *p53* aberrant expression, the UFT group showed significantly better prognosis than the Control group (95.2% and 74.3%, respectively, $P = 0.022$, Figure 2a), demonstrating that UFT administration could improve the postoperative prognosis. In patients with *p53* aberrant expression, however, there was no significant difference in the prognosis between the UFT group and the Control group (55.8% and 62.8%, respectively, $P = 0.834$, Figure 2b), demonstrating that UFT administration was not effective in such cases. In contrast, there was no difference demonstrated in the efficacy of the UFT administration between patients with *bcl-2* expression and those without *bcl-2* expression, showing the efficacy of UFT was not influenced by the status of *bcl-2* (Table 5).

Concerning AI and the efficacy of UFT, in patients with lower AI, there was no significant difference in the prognosis between the UFT group and the Control group (5-year survival rates: 74.6% and 71.6%, respectively, $P = 0.592$, Figure 3a), showing that UFT administration was not effective in lower-AI patients. On the other hand, in patients with higher AI, the UFT group showed significantly better prognosis than the Control group (5-year survival rates: 83.3% and 67.6%, respectively, $P = 0.039$, Figure 3b), suggesting that UFT administration was effective in higher-AI patients.

To confirm the efficacy of UFT administration in patients without *p53* expression and those with higher AI, multivariate analysis was performed among each patient-group. As a result, UFT administration proved to be an independent prognostic factor to improve the prognosis among patients without *p53* aberrant expression ($P = 0.030$, relative risk (RR) and the 95% confidence interval (CI): 0.094 (0.011–0.799)), whereas not among patients with *p53* aberrant expression ($P = 0.830$, RR and the 95% CI: 1.107 (0.439–2.787)). Similarly, UFT administration proved to be an independent prognostic factor to improve the prognosis among higher-AI ($P = 0.032$, RR and the 95% CI: 0.255 (0.068–0.900)), whereas not among lower-AI patients ($P = 0.311$, RR and the 95% CI: 0.599 (0.214–1.635)).

### DISCUSSION

In the present study, it was suggested that efficacy of UFT administration as a postoperative adjuvant therapy for NSCLC might be influenced by incidence of apoptosis (AI) and status of *p53* regulating apoptosis. UFT is a 5-FU derivative chemotherapeutic agent developed in Japan, and has been widely used in Japan

**Table 4** Multivariate analysis of prognostic factors (Cox's proportional hazard model)

| Prognostic factors | β | *P* value | Relative hazard (95% confidence interval) |
|---|---|---|---|
| Gender (Male/Female) | 0.049 | 0.915 | 1.050 (0.432–2.550) |
| Age | 0.023 | 0.276 | 1.023 (0.982–1.065) |
| Performance status (0/1) | 0.528 | 0.256 | 1.695 (0.682–4.211) |
| Pathologic-T factor (1/2) | 0.084 | 0.818 | 1.088 (0.532–2.224) |
| Histology (Adenocarcinoma or not) | 0.454 | 0.254 | 1.575 (0.723–3.435) |
| *p53* aberrant expression | | | |
| (Negative/Positive) | 0.983 | 0.008 | 2.673 (1.290–5.539) |
| *bcl-2* expression | | | |
| (Negative/Positive) | 0.292 | 0.531 | 1.340 (0.537–3.345) |
| Apoptotic index (AI)[a] | | | |
| (Lower/Higher) | 0.509 | 0.186 | 1.663 (0.782–3.540) |
| Proliferative index (PI)[b] | | | |
| (Lower/Higher) | −0.748 | 0.054 | 0.473 (0.221–1.013) |
| Postoperative UFT (−/+) | −1.220 | 0.012 | 0.295 (0.115–0.761) |

[a]Lower-AI: AI < 10.9 (median AI). Higher-AI: AI ≥ 10.9 (median AI). [b]Lower-PI: PI < 44% (median PI). Higher-PI: PI ≥ 44% (median PI).

 © 2001 Cancer Research Campaign

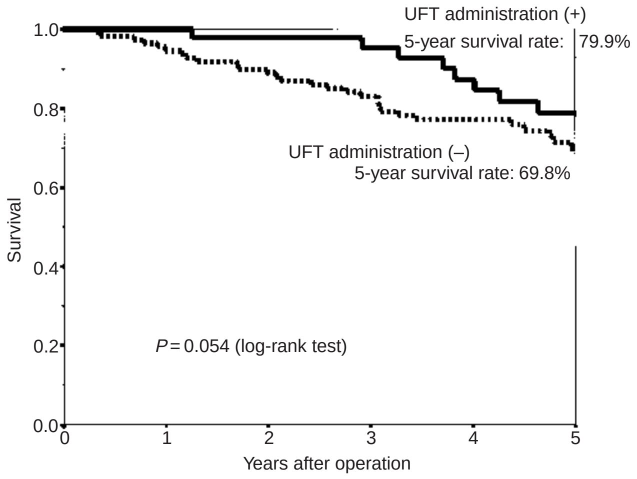

**Figure 1** Survival after complete tumour resection with lymph node dissection for p-stage I, non-small cell lung cancer (NSCLC). Comparison between patients with postoperative UFT administration and those without postoperative UFT administration

**Table 5** Postoperative survival and influence of UFT administration

| | 5-year survival rate (%) | | |
|---|---|---|---|
| | **Patients with UFT administration** | **Patients without UFT administration** | ***P* value**[a] |
| All patients | 79.9 | 69.8 | 0.054 |
| *Stratified by apoptosis and apoptosis-related gene status* | | | |
| Apoptosis index (AI)[b] | | | |
| Lower-AI | 74.6 | 71.6 | 0.592 |
| Higher-AI | 83.3 | 67.6 | 0.039 |
| *p53* status | | | |
| Aberrant expression: negative | 95.2 | 74.3 | 0.022 |
| Aberrant expression: positive | 55.8 | 62.8 | 0.834 |
| *bcl-2* status | | | |
| Expression: negative | 78.5 | 68.8 | 0.089 |
| Expression: positive | 78.8 | 75.2 | 0.873 |

[a]P-value: Comparison of patients' characteristics between patients with and without postoperative administration of UFT. [b]Lower-AI: AI < 10.9 (median AI). Higher-AI: AI ≥ 10.9 (median AI).

mainly for malignant tumours originating from the digestive system. Recently, UFT has been introduced to clinical trials conducted in the United States. Pazdur and coworkers have already reported that UFT is effective to metastatic colorectal cancer when administrated with leucovorin (Pazdur et al, 1994). Although 5-FU has been used as a basic chemotherapeutic agent for a variety of solid tumours, it has been reported that 5-FU is not effective to primary lung cancer. However, all these reports are based on results obtained by an intravenous bolus injection. Many experimental and clinical studies have revealed that 5-FU is not a dose-dependent but a time-dependent agent (Pinedo and Peter, 1988; Lokich et al, 1989). Oral administration of 5-FU and the derivatives which can continuously maintain 5-FU concentration is an extremely useful method when the pharmacological features of 5-FU are considered (Fujii et al, 1978; Ikenaka et al, 1979).

UFT is inferior to other chemotherapeutic agents such as CDDP in terms of potency of direct anticancer activity. However, these chemotherapeutic agents with potent antitumour effect have not improved postoperative survival of NSCLC. The most important factor that worsens the prognosis of completely resected NSCLC patients is high incidence of distant metastasis after operation. Recently, it has been revealed experimentally that tumour growth was suppressed with induction of apoptosis in micrometastatic lesion (Holmgren et al, 1995). We have also revealed clinically that the balance of apoptosis and proliferation of cancer cells influences on postoperative survival of NSCLC (Tanaka et al, 1999b). Postoperative oral administration of UFT maintains a certain blood concentration of 5-FU, which may accelerate apoptosis of cancer cells in micrometastatic lesions without suppression of patients' immunity, resulting in improving the postoperative prognosis. Association of the efficacy of UFT with apoptosis is supported by the results obtained in the present study. In patients with higher AI, that is patients whose cancer cells can be easily induced to apoptotic death without treatment, UFT is effective as postoperative administration of UFT can suppress recurrence by accelerating apoptosis of cancer cells. On the other hand, in patients with lower AI, that is patients whose cancer cells can not be easily induced to apoptotic death, UFT administration can not regulate micrometastases by failure to induce such cancer cells to apoptosis. This speculation is consistent with results of basic experiments demonstrating that many anticancer drugs such as CDDP and 5-FU induce cancer cells to apoptosis (Sen and D'Incaki, 1992).

The *p53* gene is an important gene that regulates apoptosis. It has been demonstrated experimentally and clinically that the antitumour effect of chemotherapeutic agents was remarkably decreased if the *p53* gene is mutated (Fujiwara et al, 1994; Lowe et al, 1994; Bergh et al, 1995). These results are consistent with our results demonstrating that the efficacy of postoperative oral administration of UFT was diminished in patients with aberrant expression of *p53*, and are reasonably understandable when the function of *p53* gene and apoptosis are considered. IHS employed in the present study to determine *p53* status is clinically useful, because it is very easy and inexpensive as well as it can be performed on paraffin-embedded slides. However, accurate detection of *p53* gene mutation can be achieved only when a complete sequence of the *p53* gene is determined (Carbone et al, 1994). In future study, correlation between efficacy of UFT and the presence of *p53* gene mutation should be examined.

The *bcl-2* gene was cloned as a gene that prolongs a cellular life by inhibiting apoptosis. Although there has been many reports on significance of *bcl-2* expression in NSCLC, a definite conclusion has not yet been established (Pezzella et al, 1993; Pastorino et al, 1997). Because the incidence of *bcl-2* expression in NSCLC is as low as about 20%, the significance of *bcl-2* expression as a prognostic factor and a predictive factor of therapeutic effect may not be important. In future, to clarify significance of *p53* aberrant expression detected with IHS and incidence of apoptosis (AI) in association with the efficacy of postoperative oral administration of UFT, prospective randomized study on the UFT administration stratified by the *p53* status and AI should be conducted.

*© 2001 Cancer Research Campaign* 

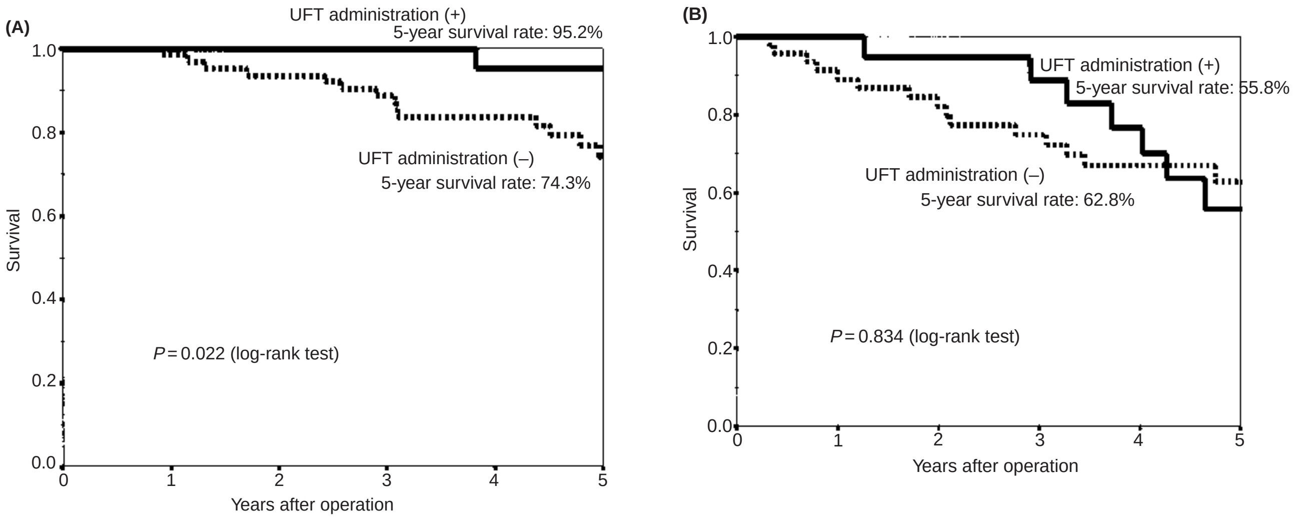

**Figure 2 (A)** Postoperative survival of patients demonstrating no aberrant *p53* expression. Comparison between patients with postoperative UFT administration and those without postoperative UFT administration. **(B)** Postoperative survival of patients demonstrating aberrant *p53* expression. Comparison between patients with postoperative UFT administration and those without postoperative UFT administration

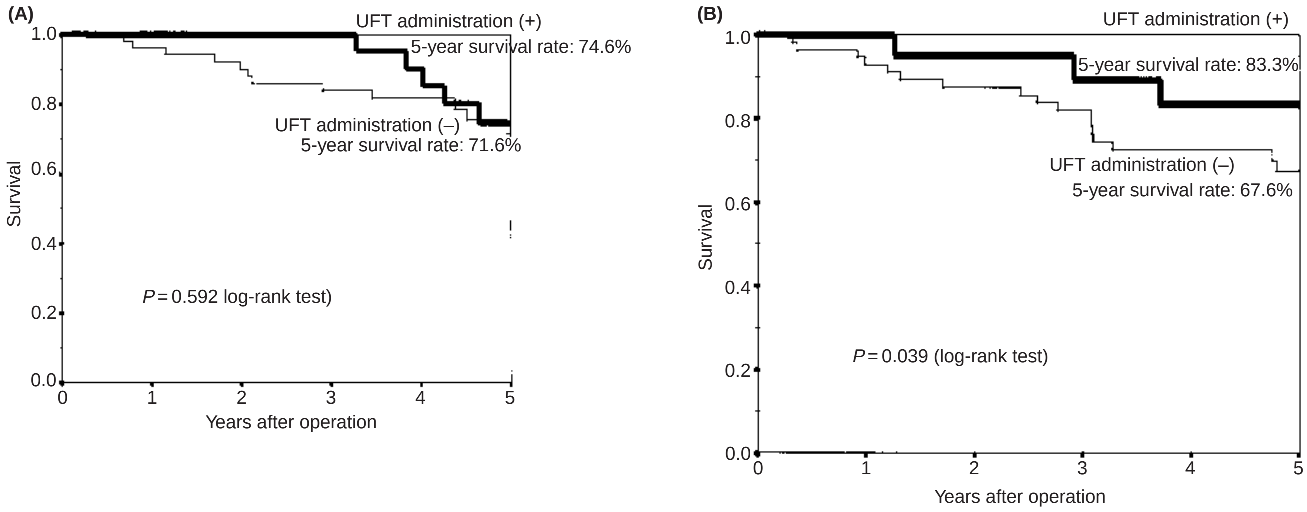

**Figure 3 (A)** Postoperative survival of patients demonstrating lower apoptotic index (AI). Comparison between patients with postoperative UFT administration and those without postoperative UFT administration. **(B)** Postoperative survival of patients demonstrating higher apoptotic index (AI). Comparison between patients with postoperative UFT administration and those without postoperative UFT administration

### ACKNOWLEDGEMENT

We thank Dr Masakazu Fukushima (Taiho Pharamaceutical Co Ltd, Saitama, Japan) for helpful discussion.

### REFERENCES

Bergh J, Norberg N, Sjogren S, Lindgre A and Holmberg L (1995) Complete sequencing of the p53 gene provides prognostic information in breast cancer patients, particularly in relation to adjuvant systemic therapy and radiotherapy. *Nature Med* **1**: 1029–1034

Carbone DP, Mitsudomi T, Chiba I, Piantadosi S, Rusch V, Nowak JA, McIntire D, Slamon D, Gazdar A and Minna J (1994) p53 immunostaining positivity is associated with reduced survival and imperfectly correlated with gene mutations in resected non-small cell lung cancer. A preliminary report of LCSG 871. *Chest* **106**: 377s–381s

Clarke AR, Purdie CA, Harrison DJ, Morris RG, Bird CC, Hooper ML and Wyllie AH (1993) Thymocyte apoptosis induced by p53-dependent and independent pathways. *Nature* **362**: 849–852

Fujii S, Ikenaka K, Fukushima M and Shirasaka T (1978) Effect of uracil and its derivatives on antitumor activity of 5-fluorouracil and 1-(2-tetrahydrofuryl)-5-fluorouracil. *Gann (Jpn J Cancer Res)* **69**: 763–772

Fujiwara T, Grim EA, Mukhopadhyay T, Zhang W, Owan-Schaub LB and Roth JA (1994) Induction of chemosensitivity in human lung cancer cells in vivo by adenovirus-mediated transfer of the wild-type p53 gene. *Cancer Res* **54**: 2287–2291

Gasparini G, Barbareschi M, Doglioni C, Palma PD, Maruri FA, Boracchi P, Bevilacqua P, Caffo O, Morelli L, Verderio P, Pezzella F and Harris AL (1995) Expression of bcl-2 protein predicts efficacy of adjuvant treatment in operable node-positive breast cancer. *Clin Cancer Res* **1**: 189–195

Holmgren L, O'Reilly MS and Folkman J (1995) Dormancy of micrometastasis: balanced proliferation and apoptosis in the presence of angiogenesis suppression. *Nature Med* **1**: 149–153.

*© 2001 Cancer Research Campaign*

Ihde D, Ball D, Arriagada R, Bathelemy N, Benner S, Bonner J, Bureau G, Crino L, Deneffe G, Emami B, Feld R, Joseph D, Paccagnella A, Rocmans P and van Houtte P (1994) Postoperative adjuvant therapy for non-small cell lung cancer: a consensus report. *Lung Cancer* **11s**: 15–17
Ikenaka K, Shirasaka T and Kitano S (1979) Effect of uracil on metabolism of 5-fluorouracil in vitro. *Gann (Jpn J Cancer Res)* **70**: 353–359
Kerr JF, Wyllie AH and Currie AR (1972) Apoptosis: a basic biological phenomenon with wide-ranging implication in tissue kinetics. *Br J Cancer* **26**: 239–257
Levine AJ, Monmand J and Finlay CA (1991) The p53 tumor suppressor gene. *Nature* **351**: 453–456
Lokich J, Ahlgren JD, Gullo JJ, Philips JA and Fryer JG (1989) A prospective randomised comparison of continuous infusion fluorouracil with a conventional bolus schedule in metastatic colorectal carcinoma: A mid-atlantic oncology program study. *J Clin Oncol* **7**: 425–442
Lowe SW, Schmitt EM, Smith SW, Osborne BA and Jacks T (1993) p53 is required for radiation-induced apoptosis in mouse thymocytes. *Nature* **362**: 847–849
Lowe SW, Bodis S, McClatchey A, Remington L, Ruley HE, Fisher DE, Housman DE and Jacks T (1994) p53 status and the efficacy of cancer therapy. *Science* **266**: 807–810
Mountain CF (1997) Revisions in the international system for staging lung cancer. *Chest* **116**: 1710–1717
Pastorino U, Andreola S, Tagliabue E, Pezzella F, Incarbone M, Sozzi G, Buyse M, Menard S, Pierotti M and Rilke F (1997) Immunocytochemical markers in stage I lung cancer: relevance to prognosis. *J Clin Oncol* **15**: 2858–2865
Pazdur R, Lassere Y, Rhodes V, Ajani JA, Sugarman SM, Patt YZ, Jones Jr. DV, Markowitz AB, Abbruzzese JL, Bready B and Levin B (1994) Phase II trial of uracil and tegafur plus oral leucovorin: effective oral regimen in the treatment of metastatic colorectal carcinoma. *J Clin Oncol* **12**: 2296–2300.
Pezzella F, Turley H, Kuzu I, Tungekar MF, Dunnill MS, Pierce CB, Harris A, Gatter KC and Mason DM (1993) Bcl-2 protein in non-small cell lung carcinoma. *N Eng J Med* **329**: 690–694
Pinedo HM and Peter FJ (1988) Fluorouracil: biochemistry and pharmacology. *J Clin Oncol* **6**: 1653–1664
Sen S and D'Incalci M (1992) Apoptosis. Biochemical events and relevance to cancer chemotherapy. *FEBS lett* **307**: 122–127
Symonds H, Krall L, Remington L, Saenz-Robles M, Lowe S, Jacks T and Dyke TV (1994) p53-dependent apoptosis suppresses tumor growth and progression in vivo. *Cell* **78**: 703–711
Tanaka F, Miyahara R, Ohtake Y, Yanagihara K, Fukuse T, Hitomi S and Wada H (1998a) Advantage of postoperative oral administration of UFT (Tegafur and Uracil) for completely resected p-stage I-IIIa non-small cell lung cancer (NSCLC). *Eur J Cardiothorac Surg* **14**: 256–262
Tanaka F, Miyahara R, Ohtake Y, Yanagihara K, Fukuse T, Hitomi S and Wada H (1998b) Lewis Y antigen expression and postoperative survival in nonsmall cell lung cancer. *Ann Thorac Surg* **66**: 1745–1750.
Tanaka F, Yanagihara K, Ohtake Y, Miyahara R, Kawano Y, Fukuse T, Hitomi S and Wada H (1999a) p53 status predicts the efficacy of postoperative oral administration of tegafur for completely respected non-small cell lung cancer. *Jpn J Cancer Res* **90**: 432–438
Tanaka F, Kawano Y, Li M, Takata T, Miyahara R, Yanagihara K, Ohtake Y, Fukuse T and Wada H (1999b) Prognostic significance of apoptotic index in completely resected non-small cell lung cancer. *J Clin Oncol* **17**: 2728–2736
The Study Group of Adjuvant Chemotherapy for Lung Cancer (Chubu, Japan) (1995) A randomised trial of postoperative adjuvant chemotherapy in non-small cell lung cancer (the second cooperative study). Eur J Surg Oncol **21**: 69–77
Travis WD, Colby TV, Corrin B, Shimosato Y and Brambilla E (1999) *World Health Organization International Histological Classification of Tumors. Histological Typing of Lung and Pleural Tumors. Third edition*. Springer: Berlin
Wada H, Hitomi S, Teramatsu T, West Japan Study Group for Lung Cancer Surgery (1996) Adjuvant chemotherapy after complete resection in non-small-cell lung cancer. *J Clin Oncol* **14**: 1048–1054
Wada H, Miyahara R, Tanaka F and Hitomi S (1999) Postoperative adjuvant chemotherapy with PVM (Cisplatin + Vindesine + Mitomycin C) and UFT (Uracil + Tegafur) in resected stage I–II NSCLC (non-small cell lung cancer): a randomized clinical trial. West Japan Study Group for lung cancer surgery (WJSG) Eur J Cardiothorac Surg **15**: 438–443

*© 2001 Cancer Research Campaign*
